# The regulated secretory pathway in CD4^+^T cells contributes to HIV-1 transmission at the virological synapse

**DOI:** 10.1186/1742-4690-8-S2-P35

**Published:** 2011-10-03

**Authors:** Clare Jolly, Sonja Welsch, Stefanie Michor, Quentin J Sattentau

**Affiliations:** 1 Medical Division of Infection and Immunity, University College London, WC1E 6BT UK; 2 WellcomeTrust Centre for Human Genetics Division of Structural Biology, University of Oxford, OX3 7BN, UK; 3 Structural and Computational Biology Unit, European Molecular Biology Laboratory, D-69117 Heidelberg, Germany; 4 The Sir William Dunn School of Pathology, University of Oxford, OX1 3RE, UK

## 

Direct cell-cell spread of Human Immunodeficiency Virus type-1 (HIV-1) at the virological synapse (VS) is an efficient mode of dissemination between CD4^+^ T cells but the mechanisms by which HIV-1 proteins are directed towards intercellular contacts is unclear. We have used confocal microscopy and electron tomography coupled with functional virology and cell biology of primary CD4^+^T cells from normal individuals and patients with Chediak Higashi Syndrome and report that the HIV-1 VS displays a regulated secretion phenotype that shares features with polarised secretion at the T cell immunological synapse (IS). Cell-cell contact at the VS re-orientates the microtubule organising center (MTOC) and organelles within the HIV-1-infected T cell towards the engaged target T cell, concomitant with polarisation of viral proteins. Directed secretion of proteins at the T cell IS requires specialised organelles termed secretory lysosomes (SL) and we show that the HIV-1 envelope glycoprotein (Env) localises with CTLA-4 and FasL in SL-related compartments and at the VS. Finally, CD4^+^ T cells that are disabled for regulated secretion are less able to support productive cell-to-cell HIV-1 spread. We propose that HIV-1 hijacks the regulated secretory pathway of CD4^+^T cells to enhance its dissemination by cell-cell spread.

